# Par‐3 family proteins in cell polarity & adhesion

**DOI:** 10.1111/febs.15754

**Published:** 2021-03-03

**Authors:** Barry J. Thompson

**Affiliations:** ^1^ ACRF Department of Cancer Biology & Therapeutics John Curtin School of Medical Research The Australian National University Canberra ACT Australia

**Keywords:** cell adhesion, E‐cadherin, epithelia, neuroblast, polarity, stem cell

## Abstract

The Par‐3/Baz family of polarity determinants is highly conserved across metazoans and includes *C. elegans* PAR‐3, *Drosophila* Bazooka (Baz), human Par‐3 (PARD3), and human Par‐3‐like (PARD3B). The *C. elegans* PAR‐3 protein localises to the anterior pole of asymmetrically dividing zygotes with cell division cycle 42 (CDC42), atypical protein kinase C (aPKC), and PAR‐6. The same *C. elegans* ‘PAR complex’ can also localise in an apical ring in epithelial cells. *Drosophila* Baz localises to the apical pole of asymmetrically dividing neuroblasts with Cdc42‐aPKC‐Par6, while in epithelial cells localises both in an apical ring with Cdc42‐aPKC‐Par6 and with E‐cadherin at adherens junctions. These apical and junctional localisations have become separated in human PARD3, which is strictly apical in many epithelia, and human PARD3B, which is strictly junctional in many epithelia. We discuss the molecular basis for this fundamental difference in localisation, as well as the possible functions of Par‐3/Baz family proteins as oligomeric clustering agents at the apical domain or at adherens junctions in epithelial stem cells. The evolution of Par‐3 family proteins into distinct apical PARD3 and junctional PARD3B orthologs coincides with the emergence of stratified squamous epithelia in vertebrates, where PARD3B, but not PARD3, is strongly expressed in basal layer stem cells – which lack a typical apical domain. We speculate that PARD3B may contribute to clustering of E‐cadherin, signalling from adherens junctions via Src family kinases or mitotic spindle orientation by adherens junctions in response to mechanical forces.

## Graphical abstract

The Par‐3/Baz family of polarity determinants undergoes oligomeric clustering at the plasma membrane in a process akin to phase separation and formation of a biomolecular condensate. The roles of Par‐3/Baz family proteins in apical‐basal polarity and in cell–cell adhesion via adherens junctions are discussed.

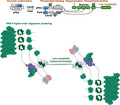

**Abbreviations**
aPKC: atypical protein kinase C
Baz: Bazooka
Cdc42: cell division cycle 42
Crb: Crumbs
PAR: partitioning defective
Scrib: scribble
Sdt: Stardust

## Introduction

Cell polarity is a fundamental feature of most eukaryotes [1, 2, 3, 4, 5, 6, 7, 8]. Pioneering genetic screens in yeast (*S. cerevisiae*) [9, 10, 11], worms (*C. elegans*) [12, 13, 14, 15] and flies (*D. melanogaster*) [16] have uncovered a set of molecular polarity determinants that function to organise the polarisation of all other molecules in the cell [17]. A central player in most eukaryotes is the cell division cycle 42 (Cdc42) GTPase [9, 10, 11], which in metazoans forms the Cdc42‐aPKC‐Par6 complex [18, 19, 20] that can be recruited to the plasma membrane by the Par‐3/Baz protein [21, 22, 23], which then forms oligomeric clusters at one pole of asymmetrically dividing stem cells, or in an apical ring in polarised epithelial cells [24, 25, 26, 27, 28, 29]. Here, we review the various roles of Par‐3/Baz family proteins in different cell types and different species, including the remarkable ability of some Par‐3/Baz family members to separate from the Cdc42‐aPKC‐Par6 complex and localise instead to adherens junctions [30, 31]. Finally, we speculate on the possible functions of Par‐3/Baz family proteins at either the apical domain or adherens junctions in both stem cells and their differentiated daughter cells.

## The discovery of PAR proteins in *C. elegans*

The partitioning defective (PAR) genes were first identified by Ken Kemphues and colleagues at Cornell University and James Priess at the MRC‐LMB in Cambridge [13]. Both Kempheus & Priess had previously trained in early *C. elegans* development in David Hirsh’s lab at the University of Colorado [14, 32, 33]. The PAR mutations affected the intracellular localisation and asymmetric partitioning of cytoplasmic ‘P granules’ during early embryonic cleavage in *C. elegans* [13]. Subsequent work from the Kemphues laboratory showed that PAR‐1 encodes a Ser/Thr kinase that is localised to the posterior of the zygote with PAR‐2, while PAR‐3 is localised to the anterior of the zygote by PAR‐6 [12, 15, 34, 35, 36]. Atypical protein kinase C (aPKC or PKC‐3) was then shown by the Ohno and Kemphues laboratories to act with PAR‐3 and PAR‐6 at the anterior pole of the cell [12, 36, 37]. Together, these results supported a model of mutual antagonism between the anterior PAR‐3, PAR‐6 & aPKC and posterior PAR‐1 & PAR‐2 polarity determinants [12], a dynamic state of polarisation which has more recently been modelled computationally [38, 39]. Recent work indicates a key role for the CDC‐42 GTPase‐activating protein (GAP) in inhibiting CDC‐42 activity at the posterior in parallel with PAR‐1 inhibition of PAR‐3 [40].

## The discovery of Cdc42 in yeast

In parallel with the characterisation of PAR proteins in *C. elegans*, Douglas Johnson, John Pringle and colleagues discovered a key role for the Cdc42 protein in cell polarity during asymmetric cell division (budding) of the yeast *S. cerevisiae* [9, 10, 41, 42]. A similar role for Cdc42 in cell polarity was later demonstrated in the fission yeast *S. pombe* [11, 43]. Subsequent work in *S. cerevisiae* has been crucial for understanding how the Cdc42 GTPase can become polarised through two distinct mechanisms: first, a positive feedback loop involving scaffolding proteins and GEF proteins that functions by clustering the complex and then activating kinases of the p21‐activating kinase (Pak) and sterile‐20 kinase (Ste20) family [5, 44]; second, a positive feedback loop involving polarised cytoskeletal trafficking of Cdc42 to the location on the membrane where it is already most concentrated [45, 46, 47]. Recent findings indicate that the ability of Cdc42 to activate Pak‐family kinases is conserved in *Drosophila* and mammalian epithelial cells [48], acting in parallel with the more famous Cdc42‐aPKC‐Par6 complex, whose discovery is described below.

## Early biochemical and genetic links between Cdc42, PAR‐6, aPKC and PAR‐3

The early genetic findings in model organisms led to experiments in mammalian epithelial cells, which suggested a possible role for Cdc42 in some aspects of epithelial polarity [49]. Biochemical experiments in mammalian cells from Tony Pawson's, Steven Martin's and Ian Macara's laboratories then decisively demonstrated direct interactions between mammalian Cdc42, Par6, aPKC (also called PKC iota or zeta in mammals) and Par3 (also called PARD3 in mammals) as well as a role for this complex at apical tight junctions [18, 19, 20, 50]. Subsequently, RNAi knockdown of the *C. elegans* Cdc42 homolog CDC‐42 demonstrated a key role in the proper localisation of PAR proteins and a direct interaction between CDC‐42 and PAR‐6 [51, 52].

## A junctional role of Bazooka in *D. melanogaster* epithelia

By 1984, genetic screens in the embryo by Eric Wieschaus & Christiane Nusslein‐Volhard had identified mutants affecting epithelial morphogenesis and cuticle formation such as *crumbs, stardust, shotgun* (encoding E‐cadherin) and *armadillo* (encoding beta‐catenin) as reviewed in Ref. [16]. Later, others sequenced these mutations and characterised the *Drosophila* Crumbs, E‐cadherin and Arm/beta‐catenin proteins [53, 54, 55, 56, 57, 58, 59, 60, 61]. A double mutant, *stardust (sdt) bazooka (baz)* was found to have a similar phenotype to *armadillo*, failing to establish a zonula adherens and disrupting formation of a polarised monolayered epithelium [23]. Removing the maternal contribution revealed an early phenotype for *baz* single mutants, suggesting a key role for *baz* in establishment of epithelial polarity and a redundant role for *baz* and *sdt* in maintenance of epithelial polarity [23].

## An apical role of Bazooka in *D. melanogaster* neuroblasts

**Fig. 1:**
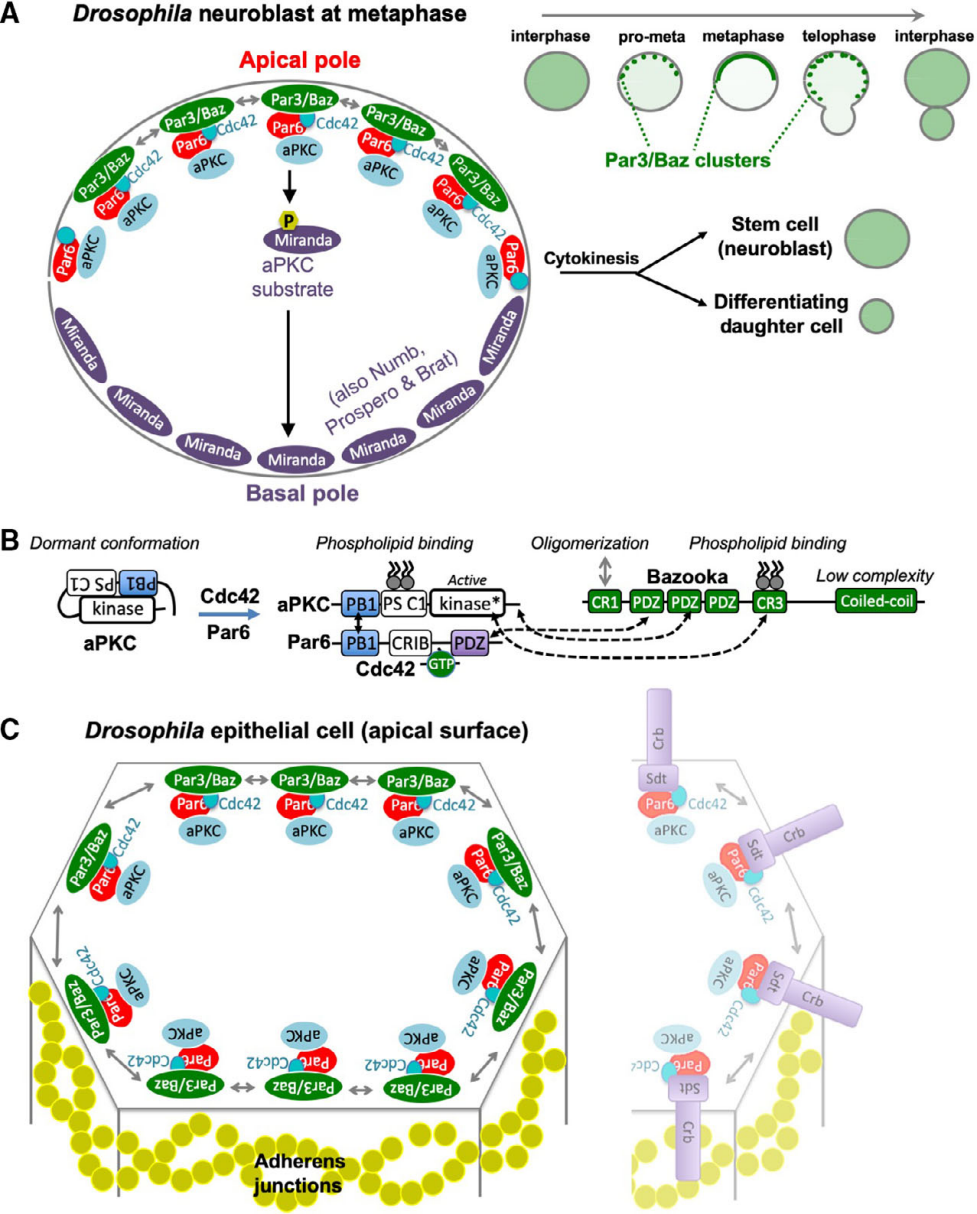
Role of Par‐3/Baz in cell polarity in neuroblasts and epithelia. (A) The *Drosophila* Par‐3 homolog Baz localises with the Cdc42‐Par6‐aPKC complex to the apical pole of neural stem cells at mitosis. Clustering of complexes via oligomeric interactions is crucial for formation of a polarised apical plasma membrane domain. (B) Protein–protein interactions upon activation of the *Drosophila* Cdc42‐Par6‐aPKC complex and its association with Baz. (C) Localisation of the Baz and Cdc42‐aPKC‐Par6 complex in an apical ring just above adherens junctions in epithelial cells (where it acts in parallel with Crumbs (Crb) protein, shown at the right).

Sequencing and characterisation of the *baz* gene revealed it to be the ortholog of *C. elegans* PAR‐3 and led to the Baz protein being found at the apical pole of asymmetrically dividing neuroblasts [27] (Fig. 1A). The neuroblast is a round stem cell that derives from epithelial cells by delamination, lacks adherens junctions, Crumbs or Stardust, and thus resembles the *C. elegans* zygote [60]. Neuroblasts divide asymmetrically to produce a self‐renewing daughter cell and a smaller differentiating daughter cell. Subsequent work showed a fundamental similarity between the molecular mechanisms of asymmetrically dividing *Drosophila* neuroblasts and *C. elegans* zygotes, both organised by polarisation of the Cdc42‐Par6‐aPKC‐Baz/Par3 complex, which then directly phosphorylates lethal giant larvae and fate determinants such as Miranda and Numb to exclude them from the apical pole [25, 28, 29, 60, 62, 63, 64, 65, 66].

Unlike in epithelial cells, polarisation of Baz to apical pole of neuroblasts only occurs during mitosis. The Aurora A cell cycle kinase plays a key role in triggering apical localisation of Baz during early pro‐metaphase [67] (Fig. 1A,B). It was proposed that Aurora A acts by phosphorylating Par6 in neuroblasts [67], but a more likely target would be a neuroblast‐specific protein that is not found in epithelial cells. The evolutionarily novel Inscuteable protein, which is uniquely expressed in neuroblasts, plays a key role in recruiting Baz apically in mitosis, and in retaining a memory of the apical pole during interphase, when Baz is cytoplasmic [68]. Notably, in specialised neuroblast‐like sensory organ precursor (SOP) cells, the entire apical‐basal axis of the cell is tilted during mitosis in a planar‐polarised fashion by interactions between Baz, Inscuteable and the Frizzled system [69, 70, 71], confirming that this spontaneously polarising system can orient itself in response to external cues.

In addition to its role in recruiting Cdc42, Par6 and aPKC, the Baz protein is also responsible for the proper orientation of the mitotic spindle in neuroblasts. Baz associates apically with Inscuteable (Insc) [27, 29, 68, 72], which in turn builds a complex of Baz, Partner of Inscuteable (Pins) and Gαi. The apical localisation of Pins‐Gαi orients the spindle at the proper angle and thereby positions the cleavage furrow, which would be randomly positioned otherwise [71, 73, 74, 75]. One report claims that mammalian Baz/Par3 (PARD3) functions similarly to recruit Pins/LGN in parallel with Gαi to orient the mitotic spindle in skin basal layer stem cells [76] – although this model remains controversial as the effect of PARD3 conditional knockout is very mild in skin (and PARD3 may not actually be expressed in skin – see below).

## Structural basis for PAR‐3/Baz clustering at the plasma membrane

**Fig. 2:**
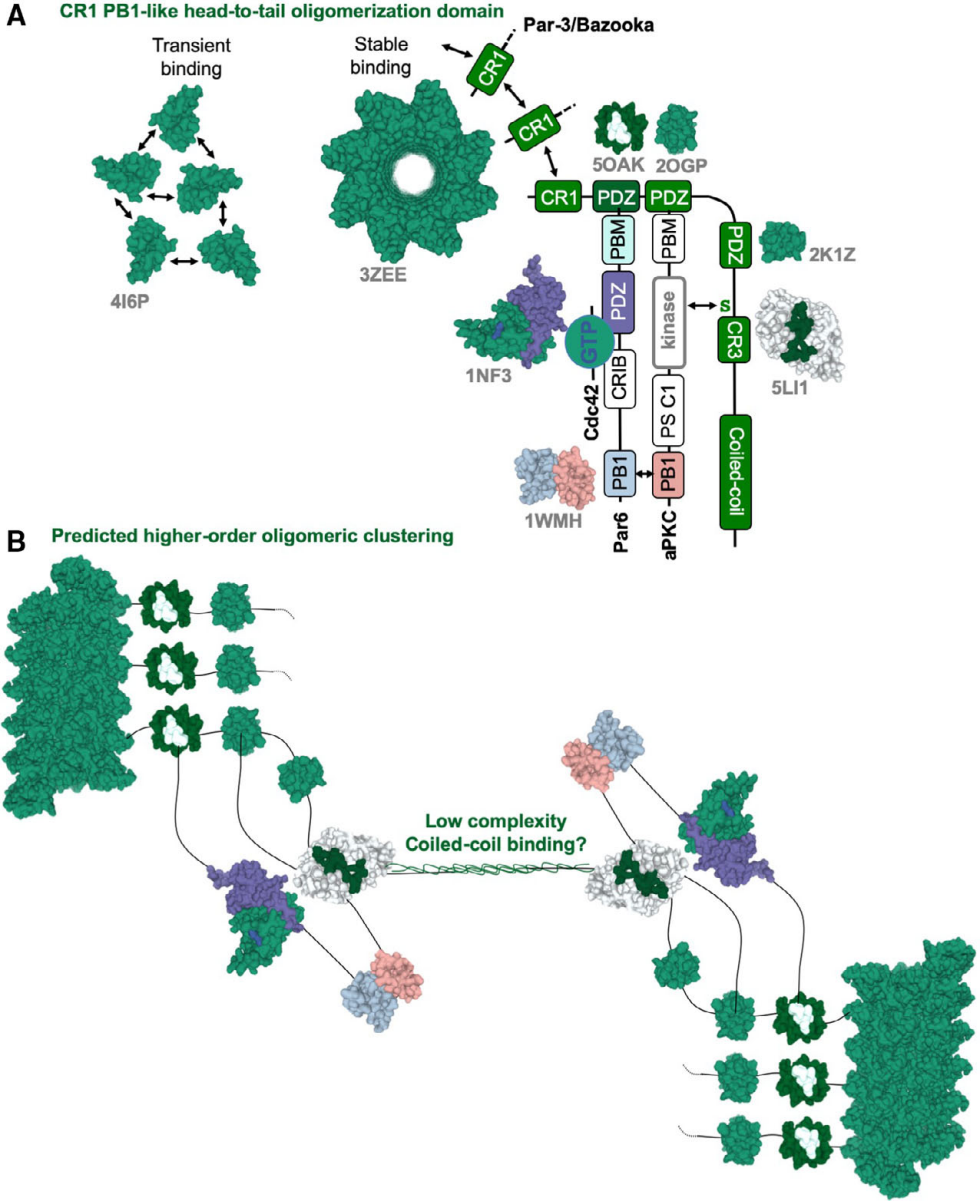
Molecular mechanisms of Par‐3/Baz clustering at the apical domain. (A) The Par‐3/Baz N‐terminal PB1‐like oligomerisation domain may either undergo dynamic and transient homomeric interactions or form a stable helical structure as observed in crystallographic studies (PDB: 3ZEE). Crystal structures of several key protein domains and their interactions have been determined in the Baz‐Par6‐aPKC‐Cdc42 complex (PDB: 5OAK, 2OGP, 1NF3, 2K1Z, 5LI1, 1WMH). (B) Higher order oligomeric clustering of Par‐3/Baz complexes may involve not only the N‐terminal PB1‐like oligomerisation domain, but also interactions between the low complexity ‘coiled‐coil’ domain (which may instead function an intrinsically disordered region) at the C‐terminus of Par‐3/Baz.

Molecularly, several mechanisms can promote clustering of PAR‐3/Baz at the plasma membrane. Firstly, head‐to‐tail oligomerisation of the N‐terminal PB1‐like domain [26, 77, 78], association of the CR3 domain with membrane lipids and interaction of Baz with a motif with aPKC’s kinase domain collectively promote clustering of Baz [26, 31, 79, 80, 81, 82, 83] (Figs 1B and Fig 2A,B). Secondly, Par6 and aPKC each have a C‐terminal PDZ‐binding motif that can bind the first and second PDZ domains in Baz, respectively (Fig. 2A,B), providing an additional molecular connection that ensures stable Baz‐aPKC‐Par6 complex formation at the apical domain [84, 85]. Thirdly, active Cdc42 binds to the Par6 semi‐CRIB domain [18, 20, 86, 87] to induce an open conformation that promotes the aPKC‐Par6 interaction (PB1:PB1 heterodimer), apical localisation of aPKC and activation of the aPKC kinase by removal of a pseudosubstrate region from the kinase cleft [88, 89] (Fig. 2A,B). The displaced pseudosubstrate region then binds to membrane lipids to further promote association of aPKC with the plasma membrane [90]. Recent evidence suggests that *Drosophila* Baz clustering at the apical pole involves phase‐separation [91], which can occur when certain proteins concentrate above a critical point and is a common characteristic of proteins with a PB1‐like head‐to‐tail oligomerisation domain [77] or intrinsically disordered regions of low complexity [92]. Since Par‐3/Baz protein has both an N‐terminal PB1‐like head‐to‐tail oligomerisation domain and C‐terminal low complexity ‘coiled‐coil’ domain, it may enable oligomers to self‐assemble into clusters that undergo phase separation (Fig. 2B).

In *Drosophila* epithelial cells, Baz clusters were observed in discrete puncta during the establishment of epithelial polarity, but form a continuous apical ring shortly thereafter (Fig. 1B). In *Drosophila* neuroblasts, at the onset of mitosis, the resulting clusters of Baz‐aPKC‐Par6‐Cdc42 rapidly enlarge and coalesce within minutes to form a uniform apical pole, which then spreads out into smaller clusters upon the mechanical enlargement of the apical domain at telophase, before returning to the cytoplasm in daughter cells [93] (Fig. 1A). In *C. elegans*, clusters of PAR‐3 were also recently observed by imaging the zygote at high resolution, with distinct CDC‐42‐Par‐6‐aPKC, PAR‐3‐PAR‐6‐aPKC and PAR‐3 homomeric clusters detected [94, 95].

## Parallel roles for Bazooka & Crumbs in *D. melanogaster* epithelia

In the polarised cuboidal/columnar epithelial cells of *Drosophila*, the Cdc42‐Par6‐aPKC‐Baz complex localises primarily in an apical ring (sometimes called the ‘subapical region’) just above the ring of adherens junctions, which it is required to position in the early embryo [23, 96, 97, 98]. Throughout embryonic development, maintenance of epithelial polarity requires Cdc42‐Par6‐aPKC [21, 22, 63, 99], but the Baz protein is only redundantly required with Crumbs‐Sdt, which becomes expressed in all epithelial cells after polarity establishment and can also recruit the Cdc42‐Par6‐aPKC complex in an apical ring [23, 100, 101] (Fig. 1B). This redundancy enables Crumbs to have other functions independent of maintaining epithelial polarity, such as acting with Spectrins to regulate the Hippo signalling pathway [102, 103], or in planar polarisation of Rho‐kinase activity during morphogenesis [104, 105]. In addition, the redundant relationship allows Baz to have an independent role at adherens junctions, including in planar polarisation of Rho‐kinase [106]. Notably, the apical localisation and planar polarity functions of *Drosophila* Baz within cuboidal/columnar epithelial cells are clearly conserved in the vertebrate PAR‐3 / Baz homologs (PARD3) in frogs [107], chickens [108, 109] and mice [110, 111, 112]

## The distinct apical and junctional pools of Bazooka in *D. melanogaster* epithelia

**Fig. 3:**
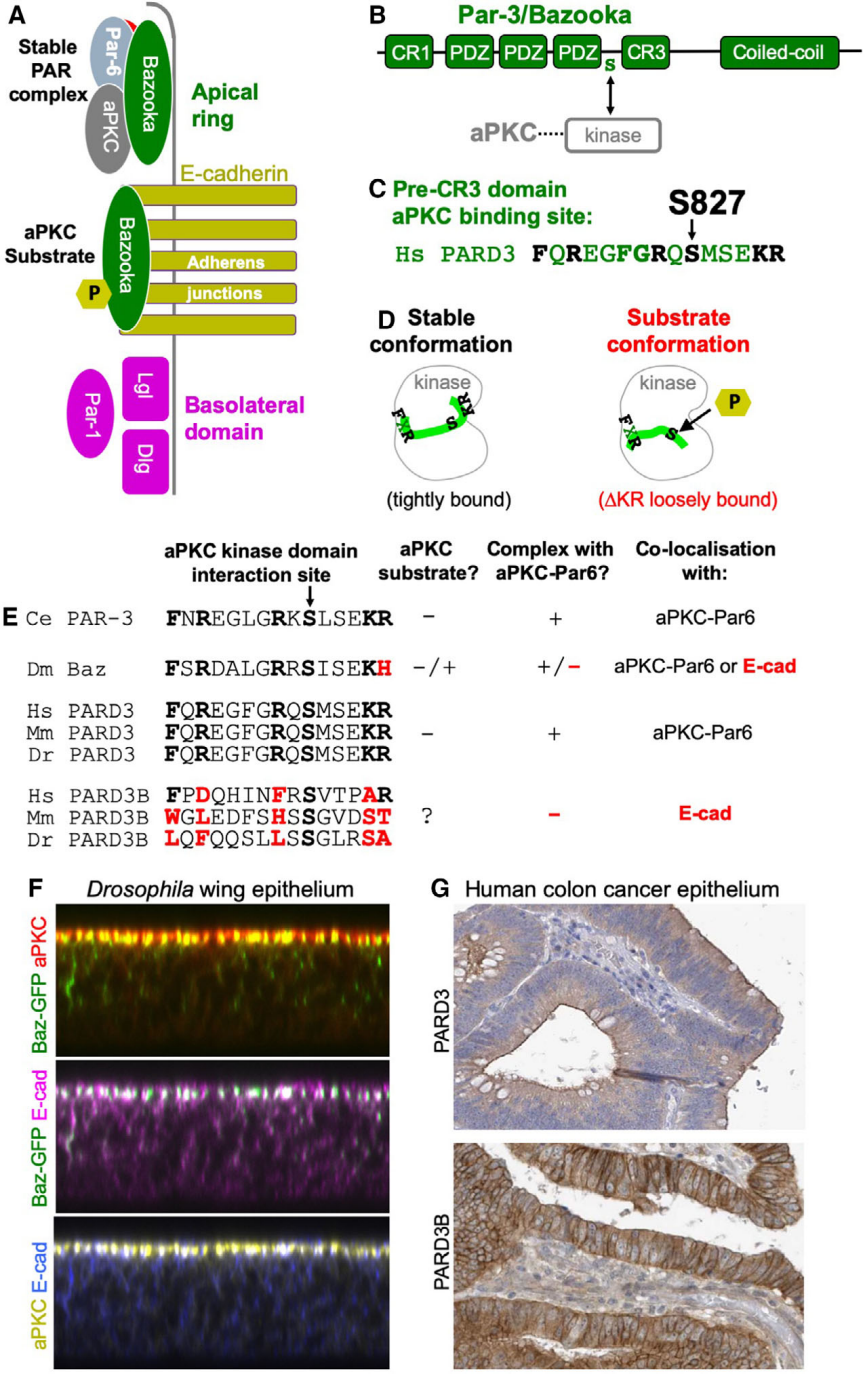
Role of Par‐3/Baz proteins at adherens junctions. (A) *Drosophila* Baz can separate from the apical PAR complex and localise to adherens junctions when phosphorylated by aPKC. (B) Diagram of interactions between Baz, Par‐6 and aPKC. (C) aPKC phosphorylation of human Par‐3 (PARD3) occurs on S827 within the aPKC interaction site. (D) A loosely bound substrate conformation may promote phosphorylation by aPKC. (E) Comparison of aPKC kinase interaction sites of Par‐3/Baz family members from different species suggests that tight binding promotes stable complex formation with aPKC‐Par6 apically, while loose binding promotes phosphorylation and dissociation from the aPKC‐Par6 complex (such that it colocalises with E‐cadherin). (F) Baz‐GFP can be detected both apically (with aPKC) and laterally (with E‐cad). (G) Human PAR‐3 (PARD3) localises apically, while PAR‐3‐like (PARD3B) localises laterally in monolayer columnar epithelial cells from colorectal cancer biopsies. Data from the www.proteinatlas.org database.

Close examination of how the *Drosophila* Baz protein localises in epithelial cells revealed that some portion of the total pool of Baz was not colocalised with Par6‐aPKC but rather colocalised with E‐cadherin upon phosphorylation by aPKC at S980 (S827 in humans) [30, 96, 97, 98, 113] (Fig. 3A‐C). Overexpression of Baz S980A disrupts the apical domain, suggesting that this construct might bind to and abnormally inhibit aPKC function [30, 31, 114]. In contrast, overexpression of Baz S980E phosphomimic simply causes localisation to adherens junctions without any epithelial disruption [31]. Thus, Baz can be in a complex with aPKC at the apical ring as long as it manages to avoid becoming a substrate for aPKC’s kinase activity, at which point it is released to junctions (Fig. 3A–C). Accordingly, there is some *in vivo* evidence for Baz inhibiting aPKC when it forms a stable complex with it [115]. The structural basis for this unusual mechanism was examined with human homologs of Baz (PARD3) and aPKC (PKC iota) [31] (Fig. 3D) and has important implications for understanding the apical vs junctional localisation of PAR‐3 / Baz family proteins.

## The distinct roles of apical PARD3 and junctional PARD3B in human epithelia

There are two orthologs of PAR‐3 / Baz in vertebrates: PARD3 (also known as PAR‐3 or PAR3) and PARD3B (also known as PAR‐3‐like or PAR3L). Vertebrate PARD3 resembles *C. elegans* PAR‐3 in its aPKC binding site, with conserved Phe‐X‐Arg (FXR) and Lys‐Arg (KR) motifs flanking the key S827 residue. In a crystal structure of the interaction between human aPKC iota kinase domain and a human PARD3 peptide, the FXR and KR motifs insert tightly into the kinase domain to form an inhibitory complex that prevents phosphorylation of S827 *in vitro* [31] (Fig. 3D,E). Another crystal structure with a peptide lacking the KR motif (which can be phosphorylated on S827) revealed a far looser binding conformation [31, 116] (Fig. 3D,E). These findings suggest that mutations in PAR‐3 / Baz family proteins that weaken the binding affinity to aPKC kinase domain will allow aPKC to phosphorylate PAR‐3 / Baz and thereby allow the protein to be localised to adherens junctions (Fig. 3E).

In the case of *Drosophila* Baz, the KR motif is altered to Lys‐His (KH), which is associated with a fractional pool of Baz being phosphorylated by aPKC and thus localising to adherens junctions with E‐cadherin [30, 31, 113] (Fig. 3E,F). In the case of vertebrate PARD3, which has intact FXR and KR motifs (Fig. 3E), antibody staining reveals localisation solely in an apical ring where aPKC is normally found, suggesting they form a stable complex [18, 19, 108, 117, 118, 119, 120] (Fig. 3G).

In the case of vertebrate PARD3B, both the FXR and KR motifs are mutated (Fig. 3E), suggesting likely disruption of the interaction with aPKC, and accordingly, antibody staining reveals localisation primarily to lateral membranes, where E‐cadherin is normally found [121] (Fig. 3F,G). Thus, PARD3 and PARD3B have diverged such that PARD3 remains tightly associated with aPKC apically, while PARD3B localises to adherens junctions. Consequently, the classical functions of PARD3 at the apical domain, including recruitment of Cdc42‐Par6‐aPKC and polarisation of membrane trafficking along the apical‐basal axis via the exocyst complex [122, 123, 124, 125, 126, 127, 128, 129, 130], appear not to be shared by PARD3B [121].

**Fig. 4:**
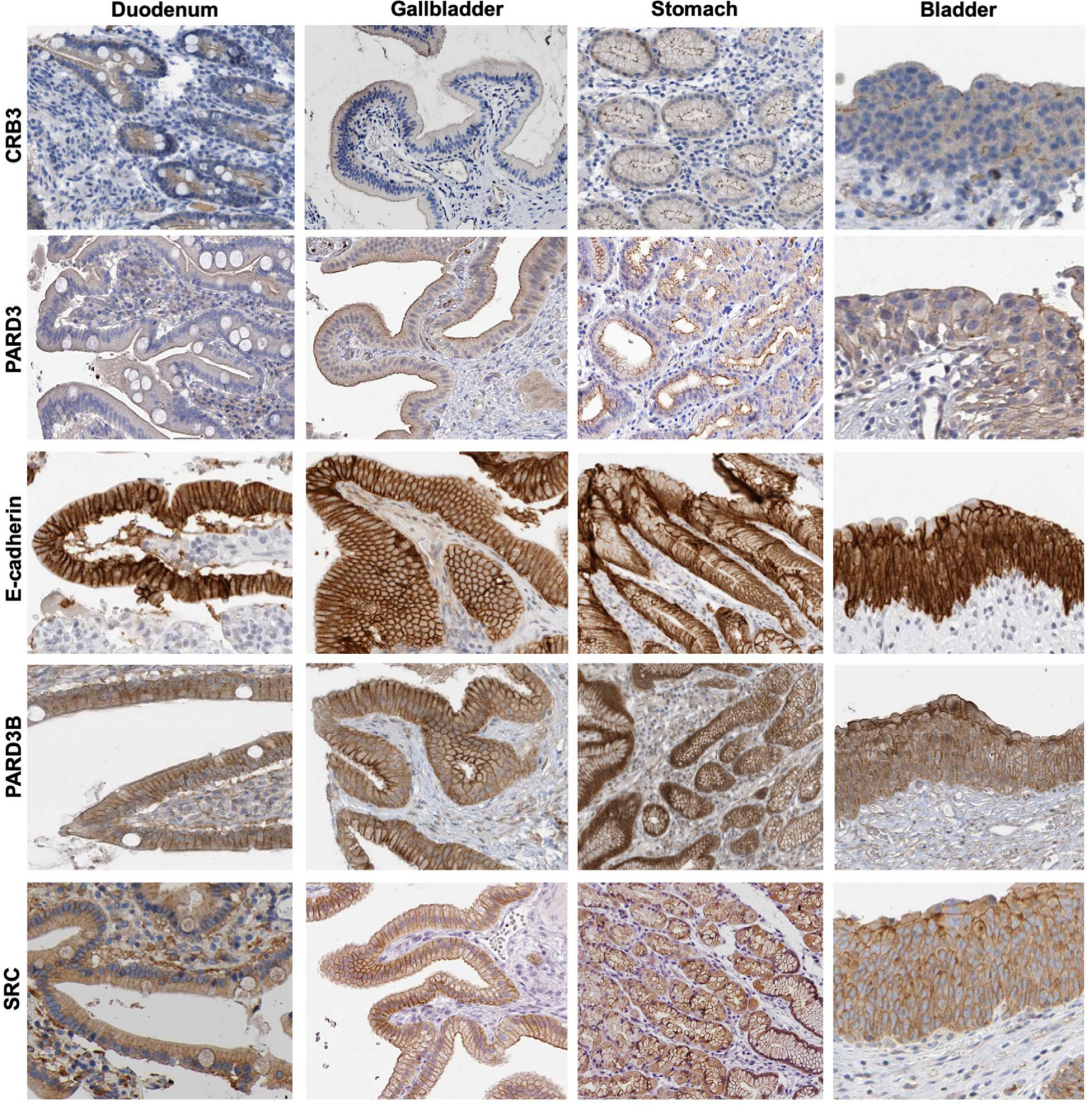
PARD3 overlaps with CRB3 apically, while PARD3B overlaps with E‐cadherin and Src laterally in monolayered columnar epithelia. Images of CRB3, PARD3, E‐cadherin, PARD3B and SRC from the www.proteinatlas.org database. Four different monolayered columnar epithelial tissues are shown, with PARD3 always apically localised and PARD3B always laterally localised with E‐cadherin and Src. Note that bladder urothelium is a monolayered pseudostratified epithelium.

**Fig. 5:**
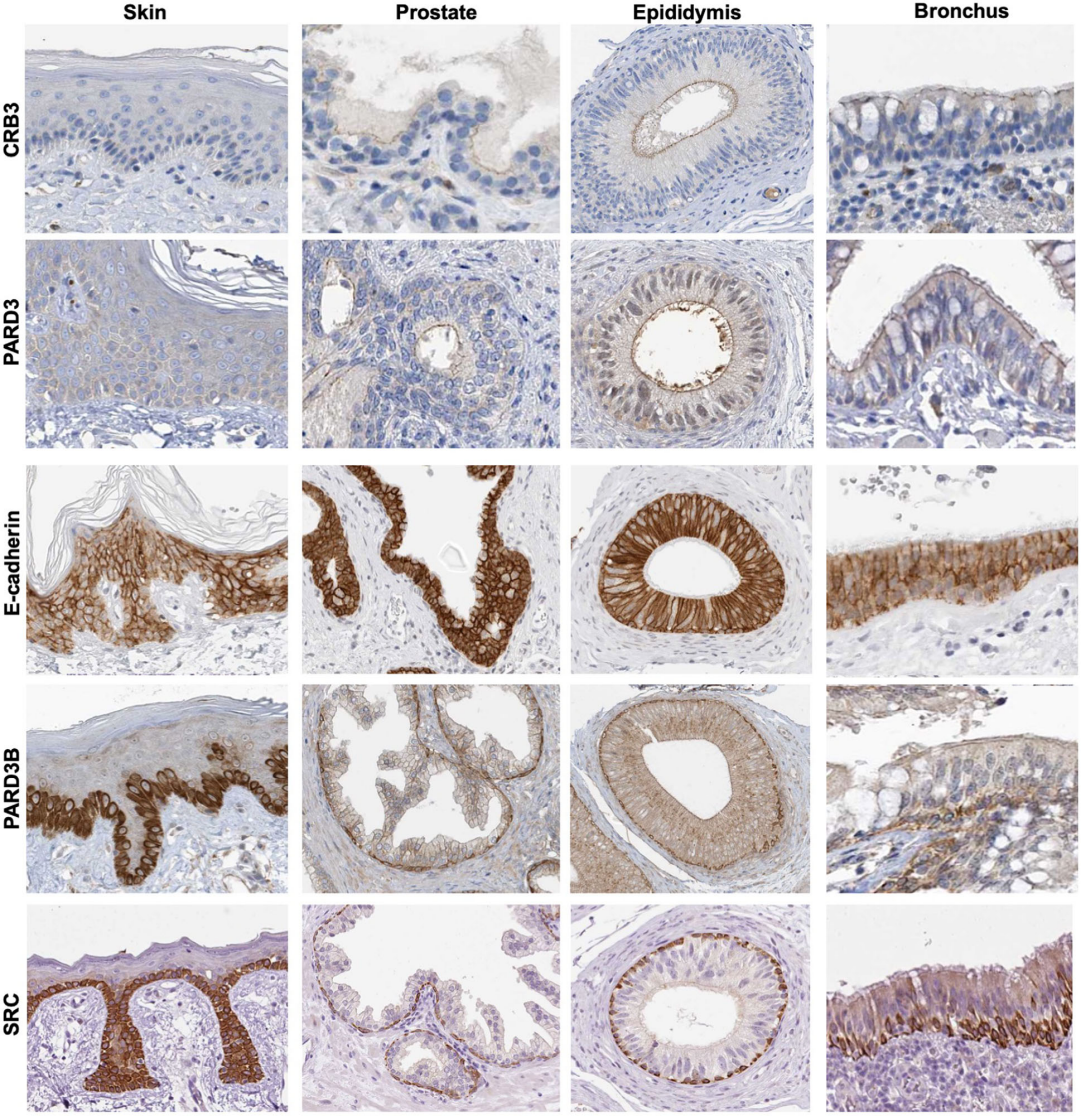
PARD3B overlaps with E‐cadherin and Src in basal layer cells of various stratified epithelia. Images of CRB3, PARD3, E‐cadherin, PARD3B and SRC from the www.proteinatlas.org database. Four different stratified epithelial tissues are shown. Note that PARD3 is not expressed in stratified squamous skin, but remains expressed and apical in stratified columnar epithelia. PARD3B and Src are restricted to the basal layer cells of skin, prostate, epididymis and bronchus.

**Fig. 6:**
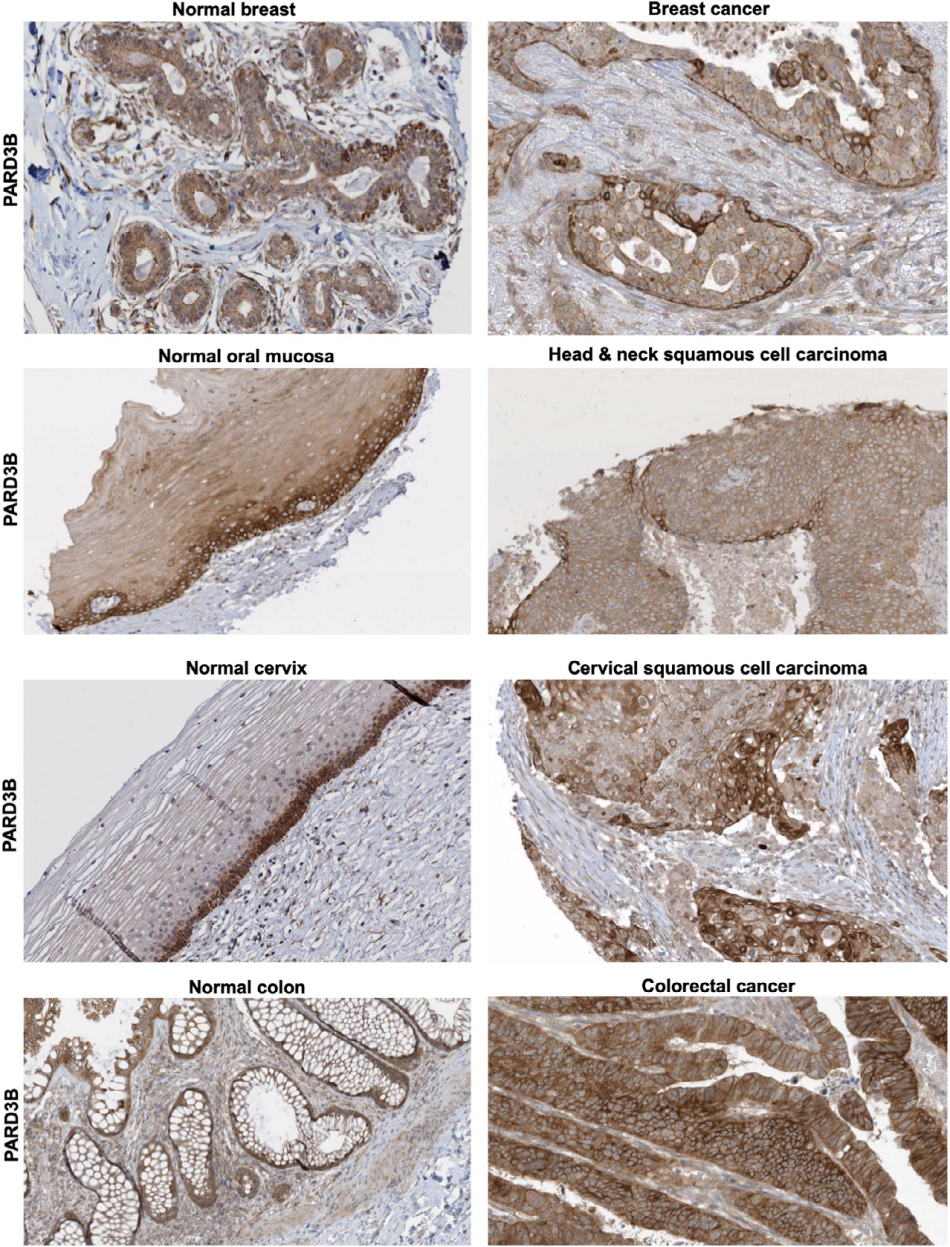
PARD3B expression in tumours arising from basal layer stem cells and monolayer epithelia. Images of PARD3B from the www.proteinatlas.org database. Four different tumour types are shown. The role of PARD3B in tumour growth and progression remains unknown.

Importantly, the apical localisation of vertebrate PARD3 and junctional localisation of PARD3B are found across a wide variety of monolayered cuboidal/columnar epithelial tissues (Fig. 4). In stratified epithelia, PARD3 is present apically in the polarised columnar cells of the bronchus, but is not found in stratified squamous epithelia where no apical domain is formed (Fig. 5). Because junctional PARD3B is strongly expressed in the basal layer of stratified epithelial cells, while E‐cadherin is ubiquitously expressed in this tissue (Fig. 5), PARD3B cannot have an essential general function in E‐cadherin trafficking to the plasma membrane or in adherens junction maintenance, but is instead likely to have a specialised function that is necessary in polarised monolayer epithelia and the basal layer of stratified epithelia – where most stem cell populations reside (Figs 4 and 5) and are the cells of origin for many epithelial cancers (Fig. 6). In an important breakthrough, Ian Macara’s laboratory showed that PARD3B is essential for stem cell maintenance in the mammary gland through a mechanism involving signal transduction rather than polarity [121]. The possible implications of this important finding for PARD3B function in stem cells are discussed below.

## Potential molecular functions of Baz and PARD3B at adherens junctions

What are the molecular functions of *Drosophila* Baz and vertebrate PARD3B at adheres junctions? In considering the possible roles of *Drosophila* Baz at junctions, it is not always easy distinguish between its role in complex with Par6‐aPKC and its solo function at adherens junctions. For example, there is evidence that Baz helps to directly position adherens junctions during polarity establishment in the early embryo [96, 97], in line with a possible role for Baz in directing polarised membrane transport and delivery of E‐cadherin to the apical/lateral junction [122, 123, 124, 125, 126, 127, 128, 129, 130]. However, the early embryonic phenotype of *baz* maternal and zygotic mutants is likely to reflect a loss of both the protein's apical and junctional functions, since Crumbs is not yet expressed at the very beginning of embryogenesis to sustain apical identity in the absence of Baz. Once Crumbs becomes expressed and helps recruit Cdc42‐Par6‐aPKC to the apical ring of epithelial cells, the unique functions of Baz at junctions can be assessed genetically. Zygotic *baz^GD21^* mutants manage to build a polarised ectodermal epithelium, but fail to normally complete morphogenetic movements such as ventral furrow formation or germ band extension [106, 131]. These defects appear to reflect a direct function of Baz at junctions during cell intercalation, which is partly driven by planar polarisation of myosin‐II‐mediated contractility to promote remodelling of adherens junctions [106, 131]. Baz itself becomes planar polarised in an opposite fashion to myosin‐II during this process, and Baz planar polarisation depends on Rho‐kinase polarisation [106, 131]. Thus, it is possible that Baz functions to antagonise excessive myosin‐II recruitment and to stabilise adherens junctions, such that strong planar activation of Rho‐kinase on the remaining sides constricts the junction and ultimately removes E‐cadherin as the junction collapses. Junctional Baz may have a similar role during photoreceptor morphogenesis [113], malpighian tubule morphogenesis [132], and segment boundary morphogenesis [133].

The role of *Drosophila* Baz during embryonic ventral furrow and dorsal fold formation is to help reposition adherens junctions in response to force as the epithelium folds [134, 135]. Baz colocalises with E‐cadherin during this repositioning, and experimentally blocking phosphorylation of Baz by Par‐1, which normally excludes Baz from basal‐lateral membranes [136], is sufficient to cause ectopic basal movement of adherens junctions along lateral membranes [134]. These findings again suggest that Baz may help stabilise adherens junctions in this context.

After the morphogenetic events of ventral furrow formation and germ‐band extension, it is unclear whether *Drosophila* Baz has any essential function in stabilising adherens junctions in most other tissues. For example, silencing Baz expression by RNAi or in mutant clones has no effect on development of wing epithelial cells or follicle cells owing to maintenance of apical identity by the Crumbs‐Sdt complex [103, 137]. This might reflect the fact that apical‐basal polarity directs formation of the apical myosin‐II contractile ring, which normally acts to maintain adherens junctions in the absence of major morphogenetic movements. Hence, Baz may be required to stabilise junctions only during specialised events where myosin‐II contractility becomes strongly polarised in order to drive tissue morphogenesis [106, 134, 138]. Notably, very strong myosin‐II‐mediated contractility during mitotic rounding is isometric, rather than polarised, and leads to downregulation of both adherens junctions and Baz in the *Drosophila* wing [139, 140] and even more dramatically in mitotic *Nematostella* epithelial cells [141] and *Xenopus* neuroepithelial cells [142].

In vertebrates, the apical and junctional roles of Baz have been split between apical PARD3 and junctional PARD3B, an evolutionary shift that coincides with the striking relocalisation of vertebrate E‐cadherin along the entire lateral membrane of epithelial cells *in vivo* (Figs 1G and 3). In *Drosophila*, E‐cadherin is clustered primarily in an apical‐lateral ring with Baz, rather than uniformly spreading along the lateral membrane (Fig. 1F), suggesting that membrane trafficking of E‐cadherin may be apically directed in this organism, possibly via polarised microtubules and the exocyst complex [123, 126, 133, 143, 144, 145, 146]. In mammalian cells, E‐cadherin appears to traffic primarily through the laterally directed AP1‐dependent pathway to the lateral membrane, [129, 147, 148, 149, 150, 151, 152, 153, 154], where it then localises with PARD3B. In *Drosophila*, E‐cadherin can also traffic via AP‐1 in nonepithelial germline cells that lack an apical‐basal axis or Baz expression [155]. In mammalian epithelial cells, apically directed trafficking via the exocyst is instead promoted by apical PARD3 [125], likely in parallel with other apical/tight‐junction determinants such as CRB‐PALS1 [128, 156, 157]. Further work is necessary to determine whether the distinct roles of apical PARD3 and junctional PARD3B contribute to the trafficking or stabilising of E‐cadherin in polarised mammalian epithelial cells.

Aside from a function in directly stabilising adherens junctions, it is also possible that *Drosophila* Baz and mammalian PARD3B might have a role in regulating signalling from adherens junctions. Src family kinases can function with Baz to recruit certain transcription factors to adherens junctions in *Drosophila* [158]. In human epithelia, Src is co‐expressed and colocalised with PARD3B at adherens junctions (Figs 3 and 4), suggesting that PARD3B might participate in E‐cadherin‐Src signalling. Interestingly, it was shown that PARD3B antagonises liver kinase B (Lkb1/STK11) signalling to help maintain mammary stem cells [121], and Src kinases have also been proposed to antagonise Lkb1 [159]. E‐cadherin‐Src signalling is thought to be mechanosensitive [160], and Src can signal to activate the YAP/TAZ family of mechanotransducers to control gene expression in the nucleus [161, 162, 163, 164] as well as acting at the cell cortex to regulate mechanical tension and stability of adherens junctions [160, 165, 166, 167, 168, 169]. Since most E‐cadherin, PARD3B and Src are localised along the entire lateral membrane of columnar cells, while the contractile ring forms at the apical‐lateral adherens junction, it may be that PARD3B and Src antagonise contractile ring formation (promoting relaxation) [170]. Both E‐cadherin and Src also have key roles in planar mitotic spindle orientation during symmetric cell divisions in epithelial cells [171, 172, 173]. In future, it will be of great interest to determine the contribution of mammalian PARD3B to E‐cadherin trafficking, adherens junction stability, E‐cadherin‐Src signalling and planar mitotic spindle orientation in various tissues and tumours (Fig. 6) using mouse conditional knockouts.

## Conclusion and perspectives

In summary, PAR‐3/Baz family proteins can have distinct roles at the apical domain (in polarity) and at adherens junctions (in signalling). In mammals, these roles are separately performed by PAR‐3 (PARD3; apical) and PAR‐3‐like (PARD3B; junctional). In *Drosophila*, there is a single PAR‐3/Baz homolog (Baz) which performs both of these functions by localising apically yet having a fractional pool that can localise to adherens junctions. In *C. elegans*, the PAR‐3 protein appears to be solely dedicated to cell polarity, rather than signalling from adherens junctions.

These differences may reflect the fundamental differences in development between invertebrates and vertebrates: particularly that *C. elegans* develops almost exclusively via the mechanism of asymmetric cell divisions in almost all lineages, while *Drosophila* development employs both asymmetric cell divisions (e.g. neuroblasts, SOPs, muscle progenitors, intestinal stem cells) and symmetric cell divisions (in epithelial tissues). There are relatively few examples of asymmetric cell divisions in mammalian development, including haematopoietic progenitors [174, 175, 176, 177, 178], T cells in contact with antigen‐presenting cells [179], muscle satellite cells [180], neural progenitors [181, 182, 183] and skin stem cells [76, 184] – and more evidence is needed for an instructive role of asymmetric division in determining cell fate through inheritance of localised determinants, rather than cell fate determination by positional information or stochastic events *after* cell division. For example, asymmetric cell division is thought to be important in neural progenitor cells, where PARD3 localises apically and is required for apical Notch signalling, which determines the fate of stem cells (high Notch) vs differentiating daughters (low Notch) [181, 182, 183]. However, PARD3 becomes transiently cytoplasmic during mitosis in the frog neuroepithelium [142], which argues that this stem cell fate decision could be made after cell division depending on whether or not a daughter cell delaminates from the epithelium and thereby loses its apical domain – in which case the orientation of the mitotic spindle and asymmetric division would not be directly responsible to fate determination. Thus, the importance of asymmetric cell division in vertebrate stem cells is still controversial.

In contrast to the asymmetric cell division model, most mammalian stem cells undergo symmetric cell divisions within monolayered columnar epithelia (Fig. 4), or as basal layer cells within stratified epithelia (Fig. 5), whose spindle orientation is organised by adherens junctions rather than apical determinants. In basal layer stem cells of stratified epithelia, there is no recognisable apical domain or polarised localisation of apical proteins (Fig. 5). Indeed, basal layer stem cells actually lack expression of PARD3 and other apical proteins altogether, and instead have strong expression of junctional PARD3B, which localises with adherens junctions around the entire plasma membrane (Fig. 5). To the extent that basal layer cells may divide asymmetrically, it is primarily orchestrated by cell shape and mechanical forces acting on adherens junctions [185, 186] rather than by the presence of an apical domain that orients the spindle [76]. Furthermore, the consequence of disrupting mitotic spindle orientation in basal layer stem cells of the skin is relatively mild [187]. Thus, further work would be necessary to establish that asymmetric division itself has an important and instructive role in basal layer stem cell fate decisions, which could instead be primarily controlled by whether daughter cells remain attached to the basement membrane *after* symmetric cell division. In polarised monolayered columnar epithelia, which always divide symmetrically in the plane of the epithelium, PARD3 and PARD3B are co‐expressed but differently localised, with PARD3 being apical and PARD3B being junctional (Fig. 3). It will be interesting to learn whether PARD3B plays a role in symmetric cell division, assisting spindle orientation by adherens junctions or influencing E‐cadherin trafficking, stability or signal transduction in response to force.

While the apical role of PAR‐3/Baz family proteins in maintaining cell polarity via the Cdc42‐Par6‐aPKC complex is well understood, much less is known about the role of these proteins at adherens junctions in either *Drosophila* or mammals. Further genetic experiments are necessary to specifically knockout the adherens junction pool of Baz in *Drosophila*, and the PARD3B protein in mice. Such experiments will shed light on the functions of these proteins at adherens junctions in both epithelial stem/progenitor cells during normal development, regeneration and in the formation of tumours.

## Conflict of interest

The authors declare no conflict of interest.
